# Lifestyle behaviors, social and economic disadvantages, and all-cause and cardiovascular mortality: results from the US National Health Interview Survey

**DOI:** 10.3389/fpubh.2024.1297060

**Published:** 2024-02-28

**Authors:** Miguel Angelo Duarte Junior, Salud Pintos Carrillo, David Martínez-Gómez, Mercedes Sotos Prieto, Fernando Rodríguez-Artalejo, Verónica Cabanas Sánchez

**Affiliations:** ^1^Department of Preventive Medicine and Public Health, School of Medicine, Universidad Autónoma de Madrid, Madrid, Spain; ^2^IMDEA Food Institute, CEI UAM+CSIC, Madrid, Spain; ^3^CIBER of Epidemiology and Public Health (CIBERESP), Madrid, Spain; ^4^Department of Environmental Health, Harvard T.H. Chan School of Public Health, Boston, MA, United States

**Keywords:** public health, health behavior, epidemiology, cohort studies, socioeconomic status

## Abstract

**Aim:**

To examine the independent relationships of lifestyle and social and economic factors with all-cause and cardiovascular disease (CVD) mortality in a large representative sample of the US adult population. Furthermore, the association between the combination of lifestyle and social and economic factors with mortality was analyzed in detail.

**Methods:**

The sample included 103,314 participants with valid records and eligible for mortality follow-up, and information on lifestyle factors and social and economic disadvantages (NHIS waves 2000, 2005, 2010, and 2015). An unhealthy lifestyle score was constructed using information on physical activity, alcohol consumption, diet, and smoking status. Social and economic disadvantages were assessed using information on education, receipt of dividends, employment, family's home, and access to private health. Information on mortality data was determined by the National Death Index records.

**Results:**

Compared with favorable lifestyle, unfavorable lifestyle was associated with higher all-cause (HR 2.07; 95% CI 1.97–2.19) and CVD (HR 1.84; 95% CI 1.68–2.02) mortality. Higher social and economic disadvantages were also associated with higher all-cause (HR 2.44; 95% CI 2.30–2.59) and CVD mortality (HR 2.44; 95% CI 2.16–2.77), compared to low social and economic disadvantages. In joint associations, participants in the high social and economic disadvantage and unfavorable lifestyle showed a greater risk of all-cause (HR 4.06; 95% CI 3.69–4.47) and CVD mortality (HR 3.98; 95% CI 3.31–4.79).

**Conclusion:**

Lifestyle and social and economic disadvantages are associated with all-cause and CVD mortality. The risk of mortality increases as the number of social and economic disadvantages and unhealthy lifestyles increases.

## 1 Introduction

Modifiable behavioral factors, such as physical activity, alcohol consumption, diet, and smoking, are directly related to health status and all-cause and cardiovascular disease (CVD) mortality (1, 2). Their impact on health could differ when considered individually or in combination (i.e., lifestyle profile) (2–4). However, social and economic factors are also involved in the complex causal system linking lifestyle and health (5). The U.S. Department of Health and Human Services stated that the conditions in the environments where people are born, live, learn, work, and get older are strong determinants of health. These conditions affect a wide range of health, functioning, and quality-of-life outcomes, and have been classified into five major domains: (i) economic stability, (ii) education access and quality, (iii) health care access and quality, (iv) neighborhood and built environment, and (v) social community context (6).

In this same vein, the American Heart Association (AHA) highlights that social determinants of health are fundamental to cardiovascular health. They even proposed a social-ecological model illustrating the interaction between social determinants of health and cardiovascular health (7). This highlight is due to the worrying cardiovascular health data in the US population. For example, around 120 million Americans currently have ≥1 forms of CVD (8). CVD is the leading cause of death in the United States (9) and worldwide (10). CVD deaths have increased from 2011 to 2017 by 9.7% (8). This also results in an economic and public health concern, as CVD mortality generates an economic burden with an estimated average annual cost of 363.4 billion dollars (11).

Social and economic disadvantages have been identified as a barrier for adopting appropriate health behaviors which, in turn, could lead to CVD and CVD mortality (1–4, 12). Thus, understanding how lifestyle and social and economic disadvantages have an impact on mortality can provide evidence that could guide public policies to act on social and economic disadvantages, especially those that are social determinants of health; and to promote healthy lifestyles among people in unfavorable social and economic situations.

Given the current complex economic situation, with increasing health status differences between social classes in the United States (13), the large number of people who still do not meet the recommendations of physical activity, alcohol, diet, and smoking (4, 14, 15), the very low prevalence of “ideal” cardiovascular health in the US population (< 1%) (7), and the need for more research on social and economic factors and cardiovascular health (7), it is important to improve our understanding of how the interaction of these factors is related with mortality outcomes. Therefore, this study aimed to examine the independent relationships of lifestyle and social and economic factors with all-cause and CVD mortality in a large representative sample of the US adult population, who participated in the National Health Interview Survey (NHIS). Furthermore, the association between the combination of lifestyle and social and economic factors with mortality was analyzed in detail.

## 2 Methods

### 2.1 Study population

The NHIS is a representative health survey conducted annually by the National Center for Health Statistics (NCHS) to assess health practices and behaviors in the US population. The target population for the NHIS consists of the civilian non-institutionalized population residing within the 50 states and the District of Columbia at the time of the interview. The study design and data collection have been previously reported (16, 17). Briefly, in the first step, approximately 35,000 cluster households are randomly selected (per year). In each household, an adult aged 18 years or older is selected to answer an interview. Interviews are conducted by Census interviewers who are appropriately trained and directed by health survey supervisors at the U.S. Census. Data are collected through computer-assisted in-person in-home interviews, with telephone follow-up when the interview cannot be conducted in person. Full details on the survey methods and procedures are available on the NHIS website (https://www.cdc.gov/nchs/nhis/).

For the present work, we selected data collected in 2000, 2005, 2010, and 2015 because complete information on the four lifestyles of interest (diet, physical activity, smoking, and alcohol intake) was only collected in those waves. Of the total US NHIS participants (*n* = 393,032), 118,357 were adults with valid records and eligible for mortality follow-up; after excluding those with missing information on lifestyle and socioeconomic factors (*n* = 15,043), the analytic sample included 103,314 participants (Supplementary Figure S1).

### 2.2 Lifestyle factors

Physical activity was estimated according to the duration and intensity of leisure-time physical activity reported by participants. Physical activity level was classified as unhealthy if it was below the World Health Organization recommendations (i.e., 150 min of moderate physical activity per week, 75 min of vigorous activity per week, or an equivalent combination of both) (18). Alcohol consumption was evaluated according to the frequency and amount of alcohol consumed. Heavy alcohol use as an unhealthy pattern was considered according to the sex-specific criteria of the National Institute on Alcohol Abuse and Alcoholism; that is, for men, consuming >4 drinks on any day or >14 drinks per week and, for women, consuming >3 drinks on any day or >7 drinks per week (19). Participants reported information on fruit and vegetable intake during the past month; when an unhealthy consumption was defined as non-daily intake of fruits and vegetables (20–22). Smoking status was categorized as never, former, or current smoker. For this lifestyle behavior, unhealthy participants were former smokers (i.e., those who had smoked at least 100 cigarettes in his or her lifetime but who had quit smoking at the time of interview) and current smokers (i.e., those who had smoked 100 cigarettes during the lifetime and currently smokes cigarettes).

For each lifestyle risk factor, we assigned 1 point. Thus, the unhealthy lifestyle score resulted from the sum of the four risk factors and ranged between 0 and 4. Participants were classified according to this score into three lifestyle categories: “favorable” (0–1 points), “intermediate” (2 points), and “unfavorable” (3–4 points).

### 2.3 Social and economic disadvantages

Five social and economic disadvantages were considered: education, receipt of dividends, employment, family's home, and access to private health. Education was classified into two different levels: (i) high school, general equivalence degree (GED) or lower, and (ii) higher than high school. The receipt of dividends was also classified into two categories: (i) reported receiving dividends from stocks, mutual funds, or net rental income from property, royalties, estates, or trusts, and (ii) reported not receiving dividends. Employment was also divided in two categories, (i) employed; those who reported working (with or without pay), (ii) not employed: those who reported be out of the labor market. Information about the family's home was also classified into two categories: (i) owned, for those who stated that the house was owned or being bought, and (ii) not owned, for those who responded that the house was rented or other arrangement. Furthermore, we also categorized cover by private health insurance into two categories: (i) claimed to have private health insurance, and (ii) did not have cover by private health insurance. Finally, to understand how these factors can impact the risk of mortality when combined, a social and economic disadvantages score was created. Low education, not receiving dividends, not employed, not owning a house, and not having a private health insurance were considered categories of social disadvantage, assigning one point to each one. Thus, social, and economic disadvantages score ranged 0 to 5 and classified as: low (0–1), medium (2, 3), and high (4, 5).

### 2.4 Ascertainment of death

Information on all-cause and CVD mortality data was determined by the NCHS (National Center for Health Statistics) based on linkage with the National Death Index (NDI) records through December 31, 2019. All-cause mortality was the primary outcome variable. Leading underlying cause of death was classified using the 10th revision of the International Statistical Classification of Diseases, Injuries, and Causes of Death (ICD-10), and CVD mortality was defined as death for diseases of heart or cerebrovascular diseases (ICD-10 codes I00–I09, I11, I13, I20–I51, I60–I69).

### 2.5 Covariates

All covariates were self-reported, and included age, sex, race/ethnicity (non-Hispanic White, non-Hispanic Black, Hispanics, other, and no response), and marital status (single, married, separated, or widowed). Moreover, participants were asked if they had ever been told by a doctor or other health professional if they had had cancer or CVD.

### 2.6 Statistical analysis

Characteristics of the study sample at baseline are presented as frequency and percentage for categorical variables, and as mean and standard deviation for continuous variables by lifestyle categories. We used Cox proportional hazard regression models to estimate the Hazard Ratios (HR) and 95% Confidence Intervals (CI) for the association of lifestyle categories (i.e., favorable, intermediate, unfavorable), individual lifestyle risk factors (i.e., physical activity, heavy alcohol drinking, diet, and smoking), and social and economic disadvantages (i.e., education, dividends from stocks, employment, family own home, and private health insurance) with all-cause and CVD mortality. The proportional hazards assumption was confirmed using the Schoenfeld residuals method. All analyses were adjusted for sex, age, race/ethnicity, marital status, cancer, and chronic CVD conditions (model 1). Moreover, to analyze for individual risk factors an additional model was created adjusting for all other individual risk factors (model 2). That is, the analyses for individual lifestyle risk factors were mutually adjusted for physical activity, excessive alcohol consumption, diet, and smoking, while analyses for individual social and economic disadvantages were mutually adjusted for education, dividends, employment, family's home, and private health insurance.

We also performed analyses stratified by social and economic disadvantages to assess the association between lifestyle categories and all-cause and CVD mortality, considering those with a favorable lifestyle as the reference category. Finally, we tested the combined association of social and economic disadvantages with all-cause and CVD mortality using the social and economic disadvantages score and lifestyle categories. For this analysis, the reference category was those with favorable lifestyle (i.e., 0–1 lifestyle risk factors) and with low social and economic disadvantages (i.e., 0–1 social and economic disadvantages). Additionally, we fitted interaction terms between lifestyle score and each social and economic factors, and between social and economic categories and each lifestyle factors in the fully adjusted models.

All analyses accounted for the complex survey design employed in NHIS by considering sample weights, and primary sampling units and stratum for variance estimation. The analyses were performed using Stata v.16.0, establishing a level of statistical significance at *p* < 0.05.

## 3 Results

Table 1 shows the baseline characteristics of participants by lifestyle categories. Participants with unfavorable lifestyle were mostly men and had lower levels of education. Moreover, they showed lower levels of physical activity, lower consumption of fruits and vegetables and higher prevalence of heavy alcohol consumption and chronic conditions (i.e., cancer, and CVD).

**Table 1:** Characteristics of study participants by lifestyle categories.

	**All (*n =* 103,314)**	**Favorable (*n =* 40,206)**	**Intermediate (*n =* 40,223)**	**Unfavorable (*n* = 22,885)**	*p*-value^*^
Age, years	48.4 (18.0)	47.1 (18.0)	49.0 (18.4)	49.8 (17.4)	< 0.001
**Sex**, ***n*** **(%)**					
Women	57,687 (55.9)	24,093 (41.7)	22,813 (39.5)	10,781 (18.6)	< 0.001
Men	45,627 (44.1)	16,113 (35.3)	17,410 (38.1)	12,104 (26.5)	
**Race/ethnicity**, ***n*** **(%)**					
Non-Hispanic white	66,437 (64.4)	26,182 (39.4)	24,658 (37.1)	15,597 (23.4)	< 0.001
Non-Hispanic black	14,462 (14.0)	4,912 (33.9)	6,353 (43.9)	3,197 (22.1)	
Hispanic	16,921 (16.4)	6,303 (37.2)	7,314 (43.2)	3,304 (19.5)	
Others	5,337 (5.2)	2,739 (51.3)	1,841 (34.5)	757 (14.1)	
**Marital status**, ***n*** **(%)**					
Married	48,026 (46.6)	20,201 (42.0)	18,134 (38.1)	9,511 (19.8)	< 0.001
Widowed/divorced/separated	29,495 (28.6)	9,674 (32.8)	11,894 (40.3)	7,927 (26.8)	
Never married	25,650 (24.8)	10,275 (40.0)	9,954 (38.8)	5,421 (21.1)	
**Education**, ***n*** **(%)**					
>High School	58,125 (56.2)	27,426 (47.1)	21,024 (36.1)	9,675 (16.6)	< 0.001
≤ High School or GED	45,189 (43.8)	12,780 (28.2)	19,199 (42.4)	13,210 (29.2)	
**Dividends from stocks/funds**, ***n*** **(%)**					
Yes	15,448 (15.0)	7,889 (51.0)	5,221 (33.8)	2,338 (15.1)	< 0.001
No	87,866 (85.0)	32,317 (36.7)	35,002 (39.8)	20,547 (23.3)	
**Employment**, ***n*** **(%)**					
Employed	62,120 (60.1)	25,728 (41.4)	23,696 (38.1)	12,696 (20.4)	< 0.001
Not employed	41,194 (39.9)	14,478 (35.1)	16,527 (40.1)	10,189 (24.7)	
**Family's home**, ***n*** **(%)**					
Owned	64,708 (62.6)	26,735 (41.3)	24,839 (38.3)	13,134 (20.3)	< 0.001
Not owned	38,606 (37.4)	13,471 (34.8)	15,384 (39.8)	9,751 (25.2)	
**Private health insurance**, ***n*** **(%)**					
Yes	64,708 (62.6)	29,295 (43.2)	25,406 (37.6)	12,775 (18.9)	< 0.001
No	38,606 (37.4)	10,911 (30.45)	14,817 (41.3)	10,110 (28.2)	
**Physical Activity**, ***n*** **(%)**					
0 min/week	38,301 (37.0)	5,742 (14.9)	18,418 (48.0)	14,141 (36.9)	< 0.001
0.01–149.99 min/week	20,230 (19.5)	3,489 (17.2)	9,833 (48.6)	6,908 (34.1)	
150–300 min/week	15,439 (14.9)	10,766 (69.7)	4,115 (26.6)	558 (3.61)	
>300 min/week	29,344 (28.5)	20,209 (68.8)	7,857 (26.7)	1,278 (4.3)	
**Alcohol**, ***n*** **(%)**					
Never	23,108 (22.4)	10,215 (44.2)	10,569 (45.7)	2,324 (10.6)	< 0.001
Former	16,057 (15.6)	4,763 (29.6)	6,589 (41.0)	4,705 (29.3)	
Current non-heavy	55,998 (54.3)	24,789 (44.2)	20,982 (37.4)	10,227 (18.2)	
Current heavy	8,016 (7.77)	389 (4.8)	2,024 (25.25)	5,603 (69.9)	
**Diet**, ***n*** **(%)**					
Fruits ≥1 times/day	44,027 (42.6)	28,019 (63.6)	12,443 (28.2)	3,565 (8.1)	< 0.001
Vegetables ≥1 times/day	47,318 (45.8)	26,062 (55.0)	14,500 (30.6)	6,756 (14.2)	< 0.001
**Smoking**, ***n*** **(%)**					
Never	59,830 (58.0)	34,837 (58.2)	23,953 (40.0)	1,040 (1.74)	< 0.001
Former	23,521 (22.7)	3,873 (16.4)	9,530 (40.5)	10,118 (43.0)	
Current	19,963 (19.3)	1,496 (7.4)	6,740 (33.7)	11,727 (58.7)	
**Chronic conditions**, ***n*** **(%)**					
Cancer	8,985 (8.7)	3,259 (36.2)	3,440 (38.2)	2,286 (25.4)	< 0.001
Cardiovascular diseases	15,064 (14.6)	4,572 (30.3)	6,038 (40.0)	4,454 (29.57)	< 0.001

Alcohol status: Never (fewer than 12 drinks in lifetime), Former (more than 12 drinks in lifetime, but no drinks past year), Current non-heavy (1+ drinks past year), Current heavy (>4 drinks on a day or > 14 drinks a week for men, and > 3 drinks on a day or > 7 drinks per week for women). GED, General Equivalence Degree. ^*^Continuous variables were compared across categories of lifestyle using ANOVA; categorical variables were compared using chi-squared tests.

Overall, 9.4% of participants had zero lifestyle risk factors; 29.4%, 38.9%, 19.8%, and 2.3% had one, two, three and four lifestyle risk factors, respectively. Thus, the lifestyle index was classified as favorable, intermediate, and unfavorable for 38.9%, 39.0% and 22.1% of the participants, respectively. The prevalence of participants with low physical activity and former/current smokers decreased over the evaluated years. As well as the prevalence of participants in the unfavorable lifestyle category (Supplementary Table S1).

In social and economic disadvantages score, 5.5% of participants had zero social and economic disadvantages; 22.5%, 27.5%, 21.7%, 15.5%, and 7.1% had one, two, three, four and five social and economic disadvantages, respectively. The social and economic disadvantages categories were classified as low, medium, and high for 28.1%, 49.2%, and 22.7% of the participants, respectively. Participants in the low disadvantage category increased from 39.1% in 2000 to 45.0% in 2015 (Supplementary Table S2).

The prevalence of unhealthy lifestyle factors was higher among participants with social and economic disadvantage, except for alcohol consumption, when the prevalence of heavy alcohol consumption was higher among employed participants (Supplementary Table S3).

Table 2 shows the association of lifestyle categories and factors with all-cause and CVD mortality. During a mean follow-up of 10.4 years (inter-quartile range: 4.6 to 14.6), 15,377 all-cause deaths and 4,843 CVD deaths were ascertained. Comparing to participants in the favorable lifestyle category, those in the intermediate (HR 1.39; 95% CI 1.33–1.45) and unfavorable (HR 2.07; 95% CI 1.97–2.19) category had an increased risk of all-cause mortality. All lifestyle factors were individually associated with increased all-cause mortality, with HR ranging from 1.08 to 1.61 (model 2). In a similar way, participants in an intermediate (HR 1.42; 95% CI 1.30–1.54) and unfavorable (HR 1.84; 95% CI 1.68–2.02) lifestyle category had higher risk of CVD mortality compared to those in the favorable lifestyle risk category. Individually, low physical activity (HR 1.79; 95% CI 1.64–1.95) and smoking (former/current; HR 1.30; 95% CI 1.21–1.39) were associated with increased CVD mortality (model 2).

**Table 2:** Association of lifestyle categories and factors with all-cause and cardiovascular disease (CVD) mortality.

**All-cause mortality**	**n/deaths**	**Mortality (%)**	**Model 1 HR (95% CI)**	**Model 2 HR (95% CI)**
**Lifestyle categories** ^a^				
Favorable	40,206/4,205	10.4	1.00 (Ref)	-
Intermediate	40,223/6,264	15.5	1.39 (1.33 1.45)	-
Unfavorable	22,885/4,908	21.4	2.07 (1.97 2.19)	-
**Physical activity**				
≥150 min/week	44,783/3,775	8.4	1.00 (Ref)	1.00 (Ref)
< 150 min/week	58,531/11,602	19.8	1.64 (1.56 1.72)	1.61 (1.54 1.69)
**Alcohol**				
No heavy drinker	95,298/14,821	15.5	1.00 (Ref)	1.00 (Ref)
Heavy drinker	8,016/556	6.9	1.37 (1.24 1.52)	1.21 (1.09 1.34)
**Diet (Fruits and Vegetables)**				
≥2 times/day	31,435/5,226	16.6	1.00 (Ref)	1.00 (Ref)
< 2 times/day	71,879/10,151	14.1	1.17 (1.12 1.23)	1.08 (1.03 1.12)
**Smoking**				
Never	59,830/6,706	11.2	1.00 (Ref)	1.00 (Ref)
Former/Current	43,484/8,671	19.9	1.57 (1.52 1.63)	1.55 (1.49 1.61)
**CVD mortality**	**n/deaths**	**Mortality (%)**	**Model 1 HR (95% CI)**	**Model 2 HR (95% CI)**
**Lifestyle categories**				
Favorable	40,206/1,349	3.36	1.00 (Ref)	-
Intermediate	40,223/2,075	5.16	1.42 (1.30 1.54)	-
Unfavorable	22,885/1,419	6.20	1.84 (1.68 2.02)	-
**Physical activity**				
≥150 min/Week	44,783/1,045	2.33	1.00 (Ref)	1.00 (Ref)
< 150 min/Week	58,531/3,798	6.4	1.81 (1.67 1.97)	1.79 (1.64 1.95)
**Alcohol**				
No heavy drinker	95,298/4,720	4.9	1.00 (Ref)	1.00 (Ref)
Heavy drinker	8,016/123	1.5	1.25 (1.00 1.57)	1.14 (0.90 1.43)
**Diet (Fruits and Vegetables)**				
≥2 times/day	31,435/1,694	5.3	1.00 (Ref)	1.00 (Ref)
< 2 times/day	71,879/3,149	4.3	1.16 (1.07 1.25)	1.07 (0.99 1.15)
**Smoking**				
Never	59,830/2,308	3.8	1.00 (Ref)	1.00 (Ref)
Former/Current	43,484/2,535	5.8	1.31 (1.23 1.40)	1.30 (1.21 1.39)

Values are Hazard Ratio (HR) and 95% Confidence Intervals (CI) ^a^Lifestyle categories: Favorable 0–1 risk factors; Intermediate: 2 risk factors; Unfavorable: 3–4 risk factors. Model 1: Adjusted for sex, age, race/ethnicity, marital status, cancer, and cardiovascular disease condition. Model 2: model 1 plus lifestyle risk factors mutually adjusted.

Social and economic disadvantages were associated with increased all-cause and CVD mortality (Table 3). Comparing to participants in the low social and economic disadvantages, those in the medium (HR 1.68; 95% CI 1.60–1.77) and high (HR 2.44; 95% CI 2.30–2.59) category had increased risk of all-cause mortality. Similarly, participants in a medium (HR 1.78; 95% CI 1.59–1.98) and high (HR 2.44; 95% CI 2.16–2.77) groups had a higher risk of CVD mortality compared to those in the low social and economic disadvantages category. Individually, all social and economic disadvantages were associated with increased risks of mortality. For all-cause mortality the HR ranged from 1.17 to 1.71 and for CVD mortality from 1.13 to 1.68 (model 2).

**Table 3:** Association of social and economic categories and factors with all-cause and cardiovascular disease (CVD) mortality.

**All-cause mortality**	**n/deaths**	**Mortality (%)**	**Model 1 HR (95% CI)**	**Model 2 HR (95% CI)**
**Social and economic disadvantage categories** ^a^				
Low disadvantage	28,996/2,156	7.4	1.00 (Ref)	-
Medium disadvantage	50,869/7,734	15.2	1.68 (1.60 1.77)	-
High disadvantage	23,449/5,487	23.4	2.44 (2.30 2.59)	-
**Education**				
>High School	58,125/5,819	10.0	1.00 (Ref)	1.00 (Ref)
≤ High School	45,189/9,558	21.1	1.45 (1.39 1.51)	1.30 (1.25 1.36)
**Dividends from stocks/funds**				
Yes	15,448/2,947	19.0	1.00 (Ref)	1.00 (Ref)
No	87,866/12,430	14.1	1.39 (1.33 1.46)	1.23 (1.17 1.29)
**Employment**				
Employed	62,120/3,443	5.5	1.00 (Ref)	1.00 (Ref)
Not employed	41,194/11,934	28.9	1.89 (1.79 1.99)	1.71 (1.62 1.81)
**Family's home**				
Owned	64,708/10,699	16.5	1.00 (Ref)	1.00 (Ref)
Not owned	38,606/4,678	12.1	1.34 (1.28 1.41)	1.23 (1.17 1.29)
**Private health insurance**				
Yes	67,476/8,563	12.6	1.00 (Ref)	1.00 (Ref)
No	35,838/6,814	19.0	1.37 (1.32 1.43)	1.17 (1.12 1.22)
**CVD mortality**	**n/deaths**	**Mortality (%)**	**Model 1 HR (95% CI)**	**Model 2 HR (95% CI)**
**Social and economic disadvantage categories** ^a^				
Low disadvantage	28,996/563	1.9	1.00 (Ref)	-
Medium disadvantage	50,869/2,472	4.8	1.78 (1.59 1.98)	-
High disadvantage	23,449/1,808	7.7	2.44 (2.16 2.77)	-
**Education**				
>High School	58,125/1,712	2.9	1.00 (Ref)	1.00 (Ref)
≤ High School	45,189/3,131	6.9	1.49 (1.38 1.60)	1.33 (1.23 1.44)
**Dividends from stocks/funds**				
Yes	15,448/910	5.8	1.00 (Ref)	1.00 (Ref)
No	87,866/3,933	4.4	1.50 (1.37 1.64)	1.33 (1.21 1.46)
**Employment**				
Employed	62,120/850	1.3	1.00 (Ref)	1.00 (Ref)
Not employed	41,194/3,993	9.6	1.84 (1.65 2.04)	1.68 (1.51 1.87)
**Family's home**				
Owned	64,708/3,372	5.2	1.00 (Ref)	1.00 (Ref)
Not owned	38,606/1,471	3.8	1.31 (1.21 1.41)	1.20 (1.11 1.30)
**Private health insurance**				
Yes	67,476/2,643	3.9	1.00 (Ref)	1.00 (Ref)
No	35,838/2,200	6.1	1.30 (1.21 1.40)	1.13 (1.05 1.22)

Values are Hazard Ratio (HR) and 95% Confidence Intervals (CI) ^a^Social and economic disadvantage categories: Low: 0–1 disadvantage; Medium: 2–3 disadvantages; High: 4–5 disadvantages. Model 1: Adjusted for sex, age, race/ethnicity, marital status, cancer, and cardiovascular disease condition. Model 2: model 1 plus social and economic factors mutually adjusted.

**Figure 1:**
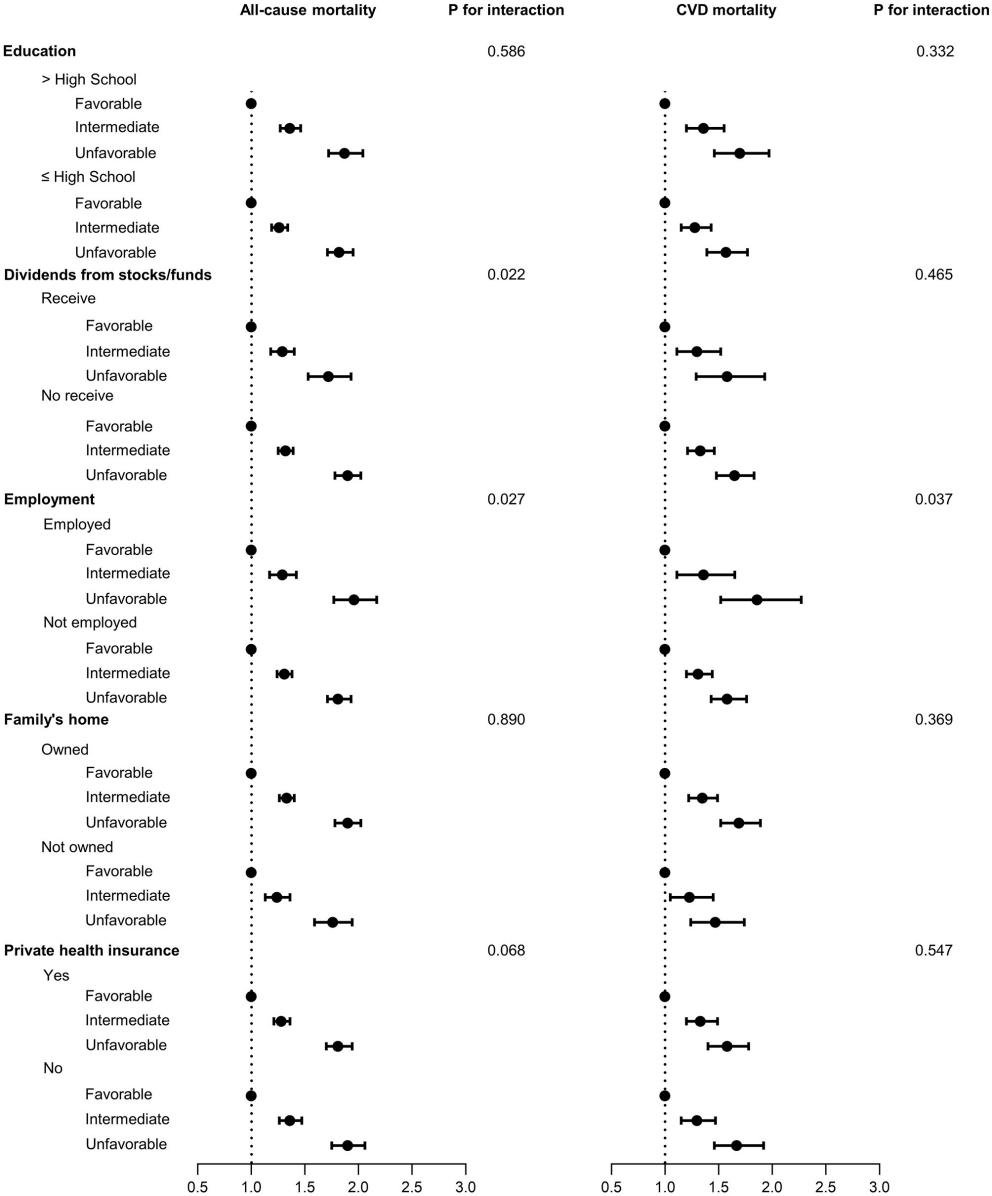
Association of lifestyle categories by social and economic factors with all-cause and cardiovascular disease (CVD) mortality. Lifestyle categories: Favorable 0–1 risk factors; Intermediate: 2 risk factors; Unfavorable: 3–4 risk factors. Model adjusted for sex, age, race/ethnicity, marital status, cancer, cardiovascular disease condition, and social and economic factors mutually adjusted.

Figure 1 presents the associations between lifestyle categories and all-cause and CVD mortality, stratified by social and economic disadvantages. Participants classified in the unfavorable lifestyle category had a higher risk of all-cause and CVD mortality when compared to intermediate and favorable categories in all sub-categories of social and economic disadvantages. However, the increased risk for intermediate and unfavorable lifestyle is very similar in the categories segmented by each social and economic disadvantage. The complete results (model 1 and model 2) of associations between lifestyle categories and all-cause and CVD mortality, stratified by social and economic disadvantages are shown in Supplementary Table S4.

**Figure 2:**
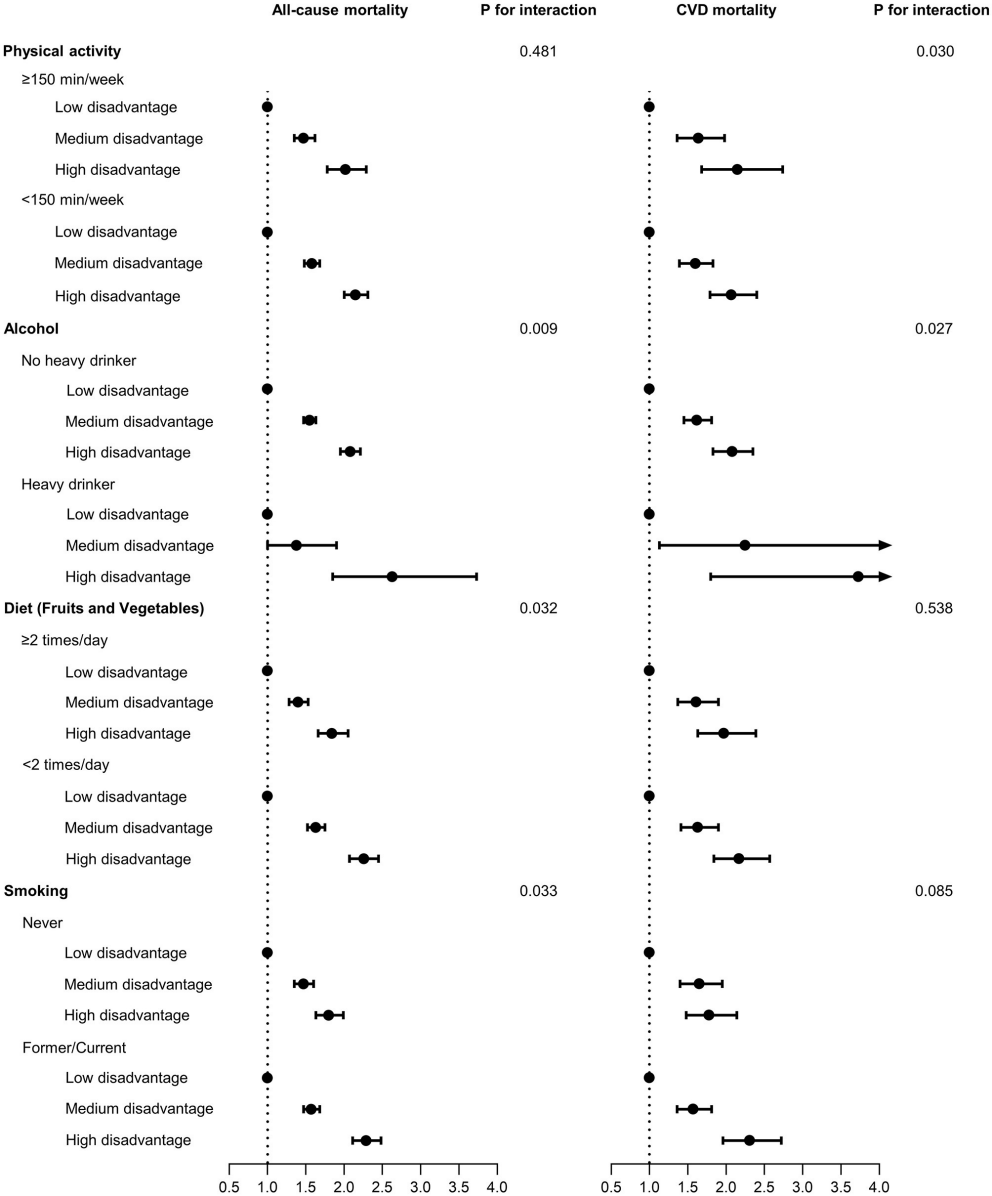
Association of social and economic disadvantages categories by lifestyle factors with all cause and cardiovascular disease (CVD) mortality. Social and economic disadvantage categories: Low: 0–1 disadvantage; Medium: 2–3 disadvantages; High: 4–5 disadvantages. Model adjusted for sex, age, race/ethnicity, marital status, cancer, cardiovascular disease condition, and lifestyle factors mutually adjusted.

Association of social and economic disadvantages categories by lifestyle factors with all-cause and CVD mortality are shown in Figure 2 and Supplementary Table S5. Participants classified in the high social and economic disadvantage category had a higher risk of all-cause and CVD mortality when compared to medium and low categories in all sub-categories of lifestyle factors. In this case, the mortality risk was higher in participants with high social and economic disadvantage category and unhealthy behavior in alcohol, diet, and smoking.

**Figure 3:**
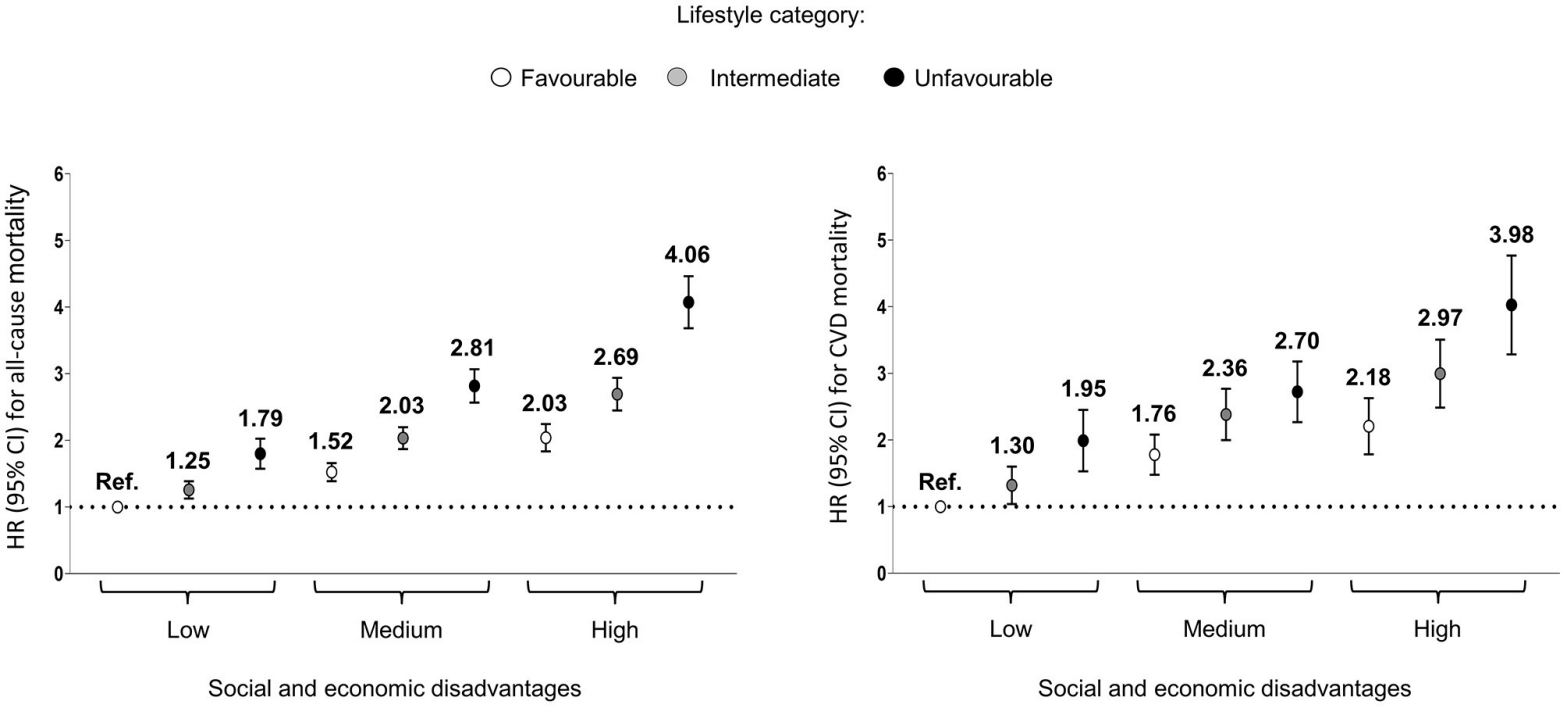
Association of combined lifestyle and social and economic disadvantage categories with risk of all-cause and cardiovascular diseases (CVD) mortality. Values are hazard ratio (HR). Lifestyle categories: Favorable 0–1 risk factors; Intermediate: 2 risk factors; Unfavorable: 3–4 risk factors. Social and economic disadvantage categories: Low: 0–1 disadvantage; Medium: 2–3 disadvantages; High: 4–5 disadvantages. Model adjusted for sex, age, race/ethnicity, marital status, cancer, and cardiovascular disease condition.

The risk of all-cause and CVD mortality increased with greater social and economic disadvantages and unfavorable lifestyle combined (Figure 3). Participants in the high social and economic disadvantage and unfavorable lifestyle shown a greater risk of all-cause (HR 4.06; 95% CI 3.69–4.47) and CVD mortality (HR 3.98; 95% CI 3.31–4.79) (Figure 3; Supplementary Table S6).

## 4 Discussion

All-cause and CVD mortality was directly related to unhealthy lifestyle factors and social and economic disadvantages. Furthermore, the risk of all-cause and CVD mortality increased with greater social and economic disadvantages and unfavorable lifestyle combined. Those in the unfavorable lifestyle category (3–4 lifestyle risk factors) and with high social and economic disadvantages (4–5 social and economic disadvantages) showed an approximately 4-fold higher risk of all-cause and CVD.

Only 9.4% of participants in our study had zero lifestyle risk factors. Previous studies also reported poor adherence to health behaviors; for example, a meta-analysis of 15 prospective studies conducted in the United States, Europe, China, and Japan, between 2004 and 2011, on lifestyle and mortality, found that only a quarter of participants adhered to all lifestyle factors (23). More recently, in a study of 0.9 million individuals from China, only 2.8% met the four health behaviors assessed (2). Therefore, our results confirmed that there is still much room for improvement in healthy behaviors at population level.

In the present study, all lifestyle risk factors were associated with a higher mortality. Specifically, when compared to engaging to the recommended PA level, low PA was associated with almost twice the risk of all-cause and CVD mortality. These results are in line with those of former studies where a strong relationship between low levels of physical activity and mortality risk has been established (24, 25). Similar results were found when considering heavy alcohol consumption as individual risk factor. Although there is constant discussion about possible benefits of moderate alcohol consumption (26), our results reinforce previous evidence that heavy alcohol consumption is indeed harmful (4, 22, 27, 28). In the same sense, our results report that a daily consumption of fruit and vegetables was associated with lower mortality risk, confirming previous results (15, 29, 30). The contribution of vitamins, minerals, antioxidants, and fiber—abundantly present in fruits and vegetables—may explain the reduction in mortality rates. It is also noteworthy that 67%−74% of the participants did not eat at least one time a day fruit and vegetables. Finally, as in previous studies (1, 22), smoking was associated with a higher risk of mortality.

Our findings showed that not complying with any or only one lifestyle factors resulted in a doubled risk of all-cause mortality compared to complying with three or all four lifestyle factors. Several reports also showed an association between adherence to lifestyle scores and mortality (2, 3, 23, 28, 31). Zhang et al. (2) pointed that complying with the four assessed lifestyle factors (non-smoking, none or moderate alcohol use, sufficient leisure time physical activity, and healthy diet) was associated with a 40% reduction of deaths from all causes and a 50% reduction of cardiovascular deaths. Similarly, other evidence suggested that a healthy lifestyle profile may be associated with an increase of around 6–9 years in life expectancy (23, 28, 31). The highest prevalence of unhealthy lifestyle factors was observed among participants with lower educational level, not receiving dividends, not employed, family's home not owned and those who did not have private health insurance. Lower prevalence of healthy lifestyle behaviors in participants with greater social disadvantage has been previously demonstrated (32).

Previous studies reported the association of social and economic factors with health outcomes and mortality (12, 32). Our results showed that participants with lower education level, no receivers of dividends from stocks/funds, not employed, family's home no owned, and no private health insurance had a higher risk of all-cause and CVD mortality, compared to each reference category. Stringhini et al. (12) reported a higher risk of mortality in people with low occupational position, as well as in people with a lower socioeconomic status, compared to people with high occupational position and high socioeconomic status. Bor et al. (13) suggest that the income inequality in the US in 1980–2015 has coincided with widening inequalities in health and longevity. The authors further related that not only do the poor have lower incomes, but also a lower life expectancy. The differences in life expectancy between the wealthiest 1% and poorest 1% were 14.6 years for men and 10.1 years for women. This reinforces and possibly explains our results on the association between social and economic disadvantages and mortality.

Seeking to understand the relationship between social and economic factors and lifestyle, Zhang et al. (3) stated that 12% of the association between socioeconomic status and mortality was explained by lifestyle factors. Petrovic et al. (33) suggested that health behaviors contribute to around 20%−26% of the associations between socioeconomic status and health outcomes. This may be an explanation for finding similar mortality associations in the stratified analyses since lifestyle explains only a limited part of the association between social and economic factors and mortality. Foster et al. (32) investigated associations between lifestyle categories and health outcomes stratified by quintiles of social deprivation in UK population. Similarly to our results, the authors found a higher mortality risk in less healthy lifestyles and for most quintiles of deprivation. However, it is not possible to identify a different risk of mortality for participants in the same lifestyle category, but in different categories of socioeconomic deprivation. This suggests that an unhealthy lifestyle may increase the risk of mortality even in subjects with better socioeconomic conditions. Similar results were found for social and economic disadvantages, advising that these factors can increase the risk of mortality, even for participants with a healthy lifestyle.

About the effect of lifestyle and social and economic factors combined on mortality, our results demonstrated that all-cause and CVD mortality was higher for participants with unfavorable lifestyle and social and economic disadvantages combined, reaching up to four times higher for these participants. Likewise, Foster et al. (32) also demonstrated a higher mortality risk in less healthy lifestyles and higher deprivation combined, mainly in participants with least healthy lifestyle and most deprived category. In this same perspective, Kollia et al. (34), in a study with 10,906 participants from a national and representative study of the English population [English Longitudinal Study of Aging (ELSA)] described those participants with lower household wealth or education, had a less healthy lifestyle. In addition, they found a higher mortality during the follow up among participants with an unhealthy lifestyle (or i.e., smoking and physical inactivity) and combination of low education and low wealth at the same time. Furthermore, it is suggested that populations with greater social vulnerability are more susceptible to the deleterious effects of unhealthy lifestyle.

Thus, our results add to this evidence, reporting how each of the different lifestyle factors and social and economic disadvantages are related to mortality risk, exploring mortality risk associated with lifestyle behaviors according to different social and economic disadvantages and vice versa, as well as the combined effect on mortality risk, using data from a representative cohort of the US population. In addition, our results suggest that intervening on lifestyle factors (i.e., physical activity, heavy alcohol consumption, diet, or smoking) and considering social and economic factors (i.e., education, economic stability, employment, family's home, and health care access) can potentially have positive effects to reduce mortality rates. However, simultaneous changes in health behaviors and social and economic circumstances can have a greater impact on health outcomes and mortality.

### 4.1 Strengths and limitations of this study

A strength of the present study is the use of data from a representative cohort of the US population, as well as the inclusion of different lifestyle indicators, considered both individually and combined as a lifestyle score. Physical activity, alcohol, diet, and smoking were included as modifiable factors, that is, health behaviors. We decided not to include biological variables, such as blood pressure or BMI, because there may be genetic and paradoxical conditions of these factors leading to a mischaracterization of the health profile. In addition, we considered diverse social and economic disadvantages, which represent different domains of social determinants of health, thus strengthening our results.

On the other hand, our study has some limitations. Information was self-reported, so recall or remember bias may have occurred. Moreover, since dietary data is collected each 5 years, we had to restrict our final analytic sample. We used the consumption of fruits and vegetables as an indicator of dietary quality because they were the available data with the highest agreement in the literature, but limiting the evaluation to only these foods could underestimate the quality of the diet. Also, information on other health behaviors such as sleep and time spent in sedentary behavior (i.e., sitting or watching TV) were not available for the selected years. In addition, only private health insurance was considered, and the public health insurance was not considered. Finally, it is important to highlight that when the lifestyle and social and economic variables were classified (0 or 1) and added to build the scores, we assumed that all health behaviors or social and economic disadvantages had an equal weight, which might not be as accurate.

## 5 Conclusion

Lifestyle, social, and economic disadvantages are associated with all-cause and CVD mortality. These associations were similar between participants with the same lifestyle category and different social and economic conditions. Therefore, even if there is a greater prevalence of unhealthy lifestyle factors among participants with social and economic disadvantages—and this should be a target population for public health interventions and policies—unhealthy lifestyle increases the risk of mortality even in individuals with better socioeconomic conditions, and social and economic disadvantages increase the risk of mortality in people with a healthy lifestyle. Furthermore, the risk of mortality increases with a greater number of social and economic disadvantages and combined unhealthy lifestyles. Therefore, to reduce mortality rates, socially vulnerable populations must be targeted by interventions that aim to promote lifestyle behaviors and increase socioeconomic equality.

## Data availability statement

The original contributions presented in the study are included in the article/Supplementary material, further inquiries can be directed to the corresponding author/s.

## Ethics statement

All NHIS content and procedures were approved by the NCHS Research Ethics Review Board. The studies were conducted in accordance with the local legislation and institutional requirements. All NHIS respondents provided oral consent prior to participation.

## Author contributions

MD: Conceptualization, Data curation, Formal analysis, Methodology, Writing—original draft, Writing—review & editing. SP: Data curation, Formal analysis, Writing—review & editing. DM-G: Conceptualization, Data curation, Formal analysis, Supervision, Writing—review & editing. MS: Conceptualization, Data curation, Supervision, Writing—review & editing. FR-A: Conceptualization, Data curation, Methodology, Supervision, Writing—review & editing. VC: Conceptualization, Data curation, Formal analysis, Methodology, Supervision, Writing—review & editing.
