# Supplementary material for: Lifestyle behaviors, social and economic disadvantages, and all-cause and cardiovascular mortality: results from the US National Health Interview Survey
Source: Front Public Health. 2024 Feb 28;12:1297060. doi: 10.3389/fpubh.2024.1297060 (PMC10933051; doi:10.3389/fpubh.2024.1297060)
Supplement: Supplementary file 1 [file Table_1.docx]

Supplementary Material

# Figure S1. Flow chart of study participants.

**
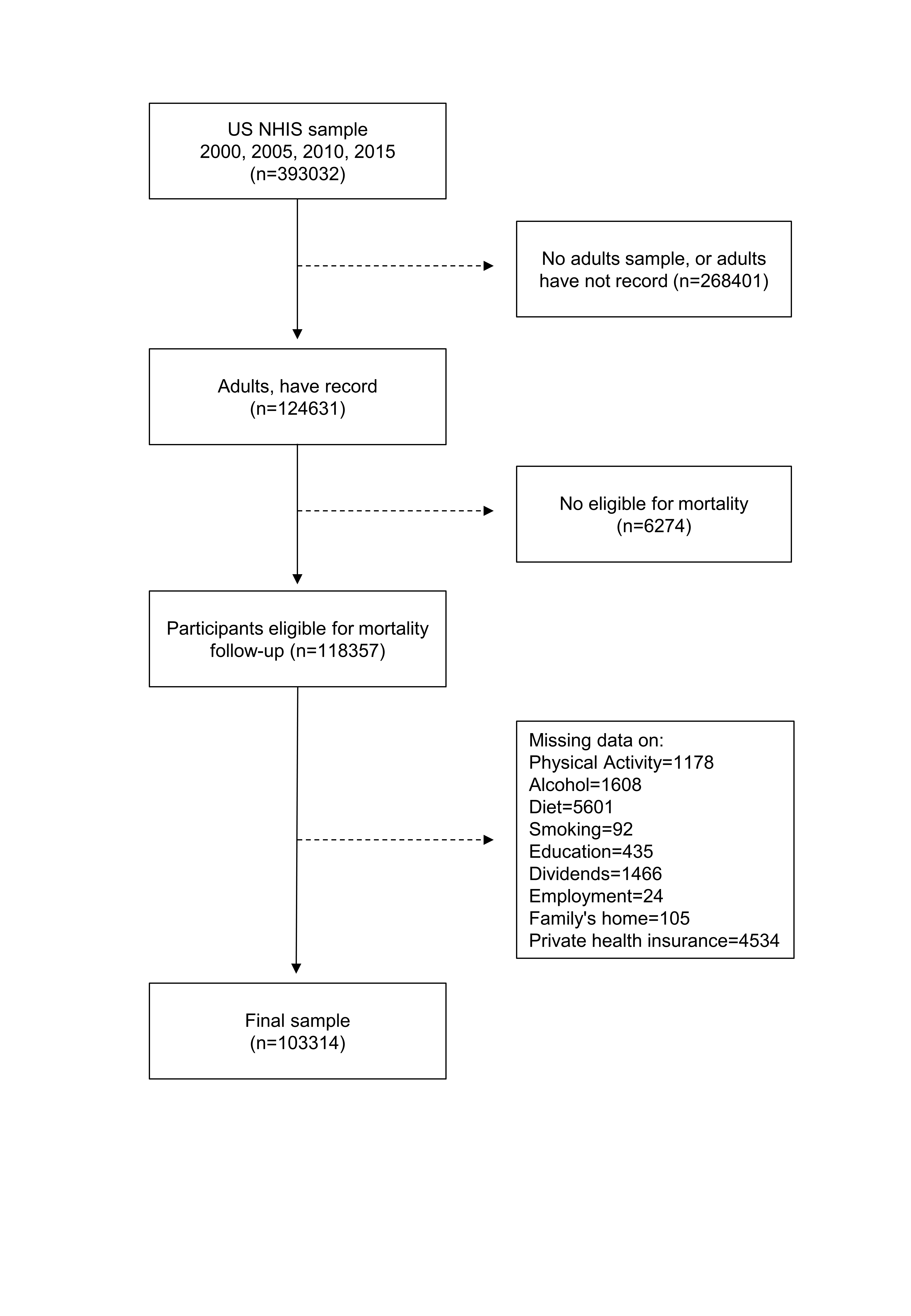
**

*NHIS identifies as a valid record the record of one person over age 18 per household who is randomly selected to complete the sample adult questionnaire.

# Table S1. Prevalence of lifestyle risk factors and categories by US NHIS wave included (2000, 2005, 2010, and 2015).

|  | 2000 | 2005 | 2010 | 2015 |
| --- | --- | --- | --- | --- |
|  | n (%) | n (%) | n (%) | n (%) |
| Individual lifestyle factors |  |  |  |  |
| Low physical activity^a^, n (%) | 13321 (55.8) | 15898 (58.7) | 13500 (52.9) | 15812 (50.8) |
| Heavy alcohol drinking^b^, n (%) | 1632 (7.0) | 2147 (8.5) | 2136 (9.3) | 2101 (7.0) |
| Unhealthy diet^c^, n (%) | 15430 (67.7) | 19298 (74.0) | 16781 (69.6) | 20370 (68.2) |
| Former/Current smoking^d^, n (%) | 10389 (45.3) | 11381 (43.2) | 9807 (41.1) | 11907 (37.1) |
| Lifestyle categories^e^ |  |  |  |  |
| Favorable, n (%) | 8854 (39.1) | 8790 (34.8) | 9602 (40.9) | 12960 (45.0) |
| Intermediate, n (%) | 8953 (38.7) | 10860 (40.7) | 9283 (37.3) | 11127 (36.4) |
| Unfavorable, n (%) | 5189 (22.1) | 6459 (24.4) | 5266 (21.8) | 5971 (18.4) |

^a^Less than 75 min of vigorous activity/week or 150 min of moderate physical activity/week, considering the WHO recommendations.

^b^Heavy drinkier was defined as consuming >4 drinks on a day or > 14 drinks a week for men, and > 3 drinks on a day or > 7 drinks per week for women.

^c^Non-daily eating of fruits and vegetables.

^d^Former smoking: Has smoked at least 100 cigarettes in his or her lifetime but who had quit smoking at the time of interview. Current smoking: have smoked 100 cigarettes during the lifetime and currently smoking cigarettes.

^e^Lifestyle categories: Favorable 0-1 risk factors; Intermediate: 2 risk factors; Unfavorable: 3-4 risk factors.

# Table S2. Prevalence of social and economic disadvantages and categories by US NHIS wave included (2000, 2005, 2010, and 2015).

|  | 2000 | 2005 | 2010 | 2015 |
| --- | --- | --- | --- | --- |
| Individual social and economic disadvantage factors |  |  |  |  |
| ≤ High School or GED, n (%) | 10747 (44.8) | 12300 (45.8) | 10481 (40.9) | 11661 (37.2) |
| No dividends from stocks/funds, n (%) | 18234 (78.0) | 22434 (85.4) | 21214 (86.6) | 25984 (87.1) |
| Not employed, n (%) | 8565 (33.8) | 9831 (34.7) | 10132 (39.1) | 12666 (38.4) |
| Not owned family house, n (%) | 7592 (25.6) | 9119 (28.2) | 9868 (32.1) | 12027 (34.1) |
| No private health insurance, n (%) | 4251 (14.1) | 9291 (31.1) | 10112 (37.0) | 12184 (36.1) |
| Social and economic disadvantage categories^e^ |  |  |  |  |
| Low disadvantage, n (%) | 7741 (39.1) | 7357 (34.8) | 6035 (40.9) | 7863 (45.0) |
| Medium disadvantage, n (%) | 11669 (38.8) | 12754 (40.7) | 11547 (37.3) | 14889 (36.5) |
| High disadvantage, n (%) | 3586 (22.1) | 5998 (24.5) | 6569 (21.8) | 7296 (18.5) |

Social and economic disadvantage categories: Low: 0-1 disadvantage; Medium: 2-3 disadvantages; High: 4-5 disadvantages

# Table S3. Prevalence of lifestyle risk factors by social and economic factors.

|  | Low physical activity^a^ | Heavy alcohol drinking^b^ | Former/Current Smoking^c^ | Unhealthy diet^d^ |
| --- | --- | --- | --- | --- |
| Education |  |  |  |  |
| > High School, n (%) | 27360 (47.0) | 3955 (6.8) | 22024 (37.8) | 38531 (66.2) |
| ≤ High School, n (%) | 31171 (68.9) | 4061 (8.9) | 21460 (47.4) | 33348 (73.8) |
| Dividends from stocks/funds |  |  |  |  |
| Receive , n (%) | 6759 (43.7) | 636 (4.1) | 6462 (41.8) | 9288 (60.1) |
| No receive, n (%) | 51772 (58.9) | 7380 (8.4) | 37022 (42.1) | 62591 (71.2) |
| Employment |  |  |  |  |
| Employed, n (%) | 31527 (50.7) | 5920 (9.5) | 24550 (39.5) | 44400 (71.4) |
| Not employed, n (%) | 27004 (65.5) | 2096 (5.0) | 18934 (45.9) | 27479 (66.7) |
| Family's home |  |  |  |  |
| Owned, n (%) | 35860 (55.4) | 3703 (5.7) | 27079 (41.8) | 43462 (67.1) |
| Not owned, n (%) | 22671 (58.7) | 4313 (11.1) | 16405 (42.4) | 28417 (73.6) |
| Private health insurance |  |  |  |  |
| Yes, n (%) | 34530 (51.1) | 4949 (7.3) | 26627 (39.4) | 45998 (68.1) |
| No, n (%) | 24001 (66.9) | 3067 (8.5) | 16857 (47.0) | 25891 (72.2) |

^a^Less than 75 min of vigorous activity/week or 150 min of moderate physical activity/week, considering the WHO recommendations.

^b^Heavy drinkier was defined as consuming >4 drinks on a day or > 14 drinks a week for men, and > 3 drinks on a day or > 7 drinks per week for women.

^c^Non-daily eating of fruits and vegetables.

^d^Former smoking: Has smoked at least 100 cigarettes in his or her lifetime but who had quit smoking at the time of interview. Current smoking: have smoked 100 cigarettes during the lifetime and currently smoking cigarettes.

# Table S4. Association of lifestyle categories by social and economic factors with all-cause and cardiovascular disease (CVD) mortality.

|  | All-cause mortality | | | | CVD mortality | | | |
| --- | --- | --- | --- | --- | --- | --- | --- | --- |
|  | n/cases | Model 1  HR (95% CI) | Model 2  HR (95% CI) | P for interaction | n/cases | Model 1  HR (95% CI) | Model 2  HR (95% CI) | P for interaction |
| Education |  |  |  | 0.586 |  |  |  | 0.332 |
| > High School |  |  |  |  |  |  |  |  |
| Favorable | 27426/1986 | 1.00 (Ref) | 1.00 (Ref) |  | 27426/589 | 1.00 (Ref) | 1.00 (Ref) |  |
| Intermediate | 21024/2279 | 1.40 (1.30 1.49) | 1.36 (1.27 1.46) |  | 21024/691 | 1.41 (1.24 1.59) | 1.36 (1.20 1.55) |  |
| Unfavorable | 9675/1554 | 2.00 (1.83 2.17) | 1.87 (1.72 2.04) |  | 9675/432 | 1.82 (1.57 2.11) | 1.70 (1.46 1.97) |  |
| ≤ High School |  |  |  |  |  |  |  |  |
| Favorable | 12780/2219 | 1.00 (Ref) | 1.00 (Ref) |  | 12780/760 | 1.00 (Ref) | 1.00 (Ref) |  |
| Intermediate | 19199/3985 | 1.29 (1.21 1.37) | 1.26 (1.19 1.34) |  | 19199/1384 | 1.31 (1.18 1.46) | 1.28 (1.15 1.43) |  |
| Unfavorable | 13210/3354 | 1.88 (1.77 2.01) | 1.82 (1.71 1.95) |  | 13210/987 | 1.63 (1.45 1.83) | 1.57 (1.39 1.77) |  |
| Dividends from stocks/funds |  |  |  | 0.022 |  |  |  | 0.465 |
| Receive |  |  |  |  |  |  |  |  |
| Favorable | 7889/1156 | 1.00 (Ref) | 1.00 (Ref) |  | 7889/359 | 1.00 (Ref) | 1.00 (Ref) |  |
| Intermediate | 5221/1128 | 1.31 (1.20 1.43) | 1.29 (1.18 1.40) |  | 5221/354 | 1.33 (1.13 1.56) | 1.30 (1.11 1.52) |  |
| Unfavorable | 2338/663 | 1.79 (1.59 2.00) | 1.72 (1.53 1.93) |  | 2338/197 | 1.65 (1.35 2.01) | 1.58 (1.29 1.93) |  |
| No receive |  |  |  |  |  |  |  |  |
| Favorable | 32317/3049 | 1.00 (Ref) | 1.00 (Ref) |  | 32317/990 | 1.00 (Ref) | 1.00 (Ref) |  |
| Intermediate | 35002/5136 | 1.38 (1.31 1.45) | 1.32 (1.25 1.39) |  | 35002/1721 | 1.39 (1.26 1.53) | 1.33 (1.21 1.46) |  |
| Unfavorable | 20547/4245 | 2.06 (1.94 2.19) | 1.90 (1.78 2.02) |  | 20547/1222 | 1.79 (1.61 1.99) | 1.65 (1.48 1.83) |  |
| Employment |  |  |  | 0.027 |  |  |  | 0.037 |
| Employed |  |  |  |  |  |  |  |  |
| Favorable | 25728/922 | 1.00 (Ref) | 1.00 (Ref) |  | 25728/216 | 1.00 (Ref) | 1.00 (Ref) |  |
| Intermediate | 23696/1325 | 1.37 (1.25 1.51) | 1.29 (1.17 1.42) |  | 23696/342 | 1.46 (1.20 1.78) | 1.36 (1.11 1.65) |  |
| Unfavorable | 12696/1196 | 2.20 (1.99 2.44) | 1.96 (1.77 2.17) |  | 12696/292 | 2.12 (1.73 2.59) | 1.86 (1.52 2.27) |  |
| Not employed |  |  |  |  |  |  |  |  |
| Favorable | 14478/3283 | 1.00 (Ref) | 1.00 (Ref) |  | 14478/1133 | 1.00 (Ref) | 1.00 (Ref) |  |
| Intermediate | 16527/4939 | 1.37 (1.31 1.45) | 1.31 (1.24 1.38) |  | 16527/1733 | 1.39 (1.27 1.52) | 1.31 (1.20 1.44) |  |
| Unfavorable | 10189/3712 | 1.96 (1.84 2.07) | 1.81 (1.71 1.93) |  | 10189/1127 | 1.73 (1.56 1.92) | 1.58 (1.43 1.76) |  |
| Family's home |  |  |  | 0.890 |  |  |  | 0.369 |
| Owned |  |  |  |  |  |  |  |  |
| Favorable | 26735/3148 | 1.00 (Ref) | 1.00 (Ref) |  | 26735/999 | 1.00 (Ref) | 1.00 (Ref) |  |
| Intermediate | 24839/4357 | 1.40 (1.33 1.47) | 1.33 (1.26 1.40) |  | 24839/1431 | 1.44 (1.30 1.58) | 1.35 (1.22 1.49) |  |
| Unfavorable | 13134/3194 | 2.06 (1.94 2.20) | 1.90 (1.78 2.02) |  | 13134/942 | 1.87 (1.68 2.09) | 1.69 (1.52 1.89) |  |
| Not owned |  |  |  |  |  |  |  |  |
| Favorable | 13471/1057 | 1.00 (Ref) | 1.00 (Ref) |  | 13471/350 | 1.00 (Ref) | 1.00 (Ref) |  |
| Intermediate | 15384/1907 | 1.31 (1.20 1.44) | 1.24 (1.13 1.36) |  | 15384/644 | 1.30 (1.11 1.52) | 1.23 (1.05 1.45) |  |
| Unfavorable | 9751/1714 | 1.95 (1.77 2.16) | 1.76 (1.59 1.94) |  | 9751/477 | 1.63 (1.38 1.92) | 1.47 (1.24 1.74) |  |
| Private health insurance |  |  |  | 0.068 |  |  |  | 0.547 |
| Yes |  |  |  |  |  |  |  |  |
| Favorable | 29295/2689 | 1.00 (Ref) | 1.00 (Ref) |  | 29295/830 | 1.00 (Ref) | 1.00 (Ref) |  |
| Intermediate | 25406/3429 | 1.34 (1.27 1.42) | 1.28 (1.21 1.36) |  | 25406/1121 | 1.42 (1.27 1.58) | 1.33 (1.20 1.49) |  |
| Unfavorable | 12775/2445 | 1.95 (1.83 2.09) | 1.81 (1.70 1.94) |  | 12775/692 | 1.74 (1.54 1.97) | 1.58 (1.40 1.78) |  |
| No |  |  |  |  |  |  |  |  |
| Favorable | 10911/1516 | 1.00 (Ref) | 1.00 (Ref) |  | 10911/519 | 1.00 (Ref) | 1.00 (Ref) |  |
| Intermediate | 14817/2835 | 1.42 (1.32 1.54) | 1.36 (1.26 1.47) |  | 14817/954 | 1.36 (1.21 1.54) | 1.30 (1.15 1.47) |  |
| Unfavorable | 10110/2463 | 2.06 (1.90 2.23) | 1.90 (1.75 2.06) |  | 10110/727 | 1.81 (1.58 2.08) | 1.67 (1.46 1.92) |  |

Values are Hazard Ratio (HR) and 95% Confidence Intervals (CI). Lifestyle categories: Favorable 0-1 risk factors; Intermediate: 2 risk factors; Unfavorable: 3-4 risk factors. Model 1: Adjusted for sex, age, race/ethnicity, marital status, cancer, and cardiovascular disease condition. Model 2: model 1 plus social and economic factors mutually adjusted.

# Table S5. Association of social and economic categories by lifestyle factors with all-cause and cardiovascular disease (CVD) mortality.

|  | All-cause mortality | | | | CVD mortality | | | |
| --- | --- | --- | --- | --- | --- | --- | --- | --- |
|  | n/cases | Model 1  HR (95% CI) | Model 2  HR (95% CI) | P for interaction | n/cases | Model 1  HR (95% CI) | Model 2  HR (95% CI) | P for interaction |
| Physical activity |  |  |  | 0.481 |  |  |  | 0.030 |
| ≥150 min/week |  |  |  |  |  |  |  |  |
| Low disadvantage | 16491/926 | 1.00 (Ref) | 1.00 (Ref) |  | 16491/213 | 1.00 (Ref) | 1.00 (Ref) |  |
| Medium disadvantage | 22045/2026 | 1.53 (1.40 1.68) | 1.47 (1.35 1.62) |  | 22045/588 | 1.70 (1.41 2.05) | 1.64 (1.36 1.98) |  |
| High disadvantage | 6247/823 | 2.16 (1.90 2.45) | 2.02 (1.78 2.29) |  | 6247/244 | 2.29 (1.79 2.92) | 2.15 (1.68 2.74) |  |
| <150 min/week |  |  |  |  |  |  |  |  |
| Low disadvantage | 12505/1230 | 1.00 (Ref) | 1.00 (Ref) |  | 12505/350 | 1.00 (Ref) | 1.00 (Ref) |  |
| Medium disadvantage | 28824/5708 | 1.63 (1.52 1.74) | 1.58 (1.48 1.68) |  | 28824/1884 | 1.62 (1.41 1.86) | 1.60 (1.39 1.83) |  |
| High disadvantage | 17202/4664 | 2.24 (2.08 2.42) | 2.15 (2.00 2.31) |  | 17202/1564 | 2.11 (1.82 2.44) | 2.07 (1.79 2.40) |  |
| Alcohol |  |  |  | 0.009 |  |  |  | 0.027 |
| No heavy drinker |  |  |  |  |  |  |  |  |
| Low disadvantage | 27455/2089 | 1.00 (Ref) | 1.00 (Ref) |  | 27455/552 | 1.00 (Ref) | 1.00 (Ref) |  |
| Medium disadvantage | 46417/7478 | 1.68 (1.59 1.77) | 1.55 (1.47 1.63) |  | 46417/2415 | 1.76 (1.57 1.97) | 1.62 (1.45 1.81) |  |
| High disadvantage | 21426/5254 | 2.40 (2.25 2.55) | 2.08 (1.95 2.21) |  | 21426/1753 | 2.40 (2.11 2.72) | 2.08 (1.83 2.35) |  |
| Heavy drinker |  |  |  |  |  |  |  |  |
| Low disadvantage | 1541/67 | 1.00 (Ref) | 1.00 (Ref) |  | 1541/11 | 1.00 (Ref) | 1.00 (Ref) |  |
| Medium disadvantage | 4452/256 | 1.48 (1.08 2.04) | 1.38 (1.00 1.90) |  | 4452/57 | 2.40 (1.18 4.88) | 2.25 (1.13 4.46) |  |
| High disadvantage | 2023/233 | 2.96 (2.08 4.20) | 2.63 (1.85 3.73) |  | 2023/55 | 4.20 (1.95 9.04) | 3.73 (1.80 7.71) |  |
| Diet (Fruits and Vegetables) |  |  |  | 0.032 |  |  |  | 0.538 |
| ≥2 times/day |  |  |  |  |  |  |  |  |
| Low disadvantage | 10166/887 | 1.00 (Ref) | 1.00 (Ref) |  | 10166/228 | 1.00 (Ref) | 1.00 (Ref) |  |
| Medium disadvantage | 15197/2746 | 1.50 (1.37 1.64) | 1.40 (1.28 1.53) |  | 15197/923 | 1.75 (1.49 2.07) | 1.61 (1.37 1.90) |  |
| High disadvantage | 6072/1593 | 2.10 (1.88 2.34) | 1.84 (1.66 2.05) |  | 6072/543 | 2.30 (1.89 2.81) | 1.97 (1.63 2.39) |  |
| <2 times/day |  |  |  |  |  |  |  |  |
| Low disadvantage | 18830/1269 | 1.00 (Ref) | 1.00 (Ref) |  | 18830/335 | 1.00 (Ref) | 1.00 (Ref) |  |
| Medium disadvantage | 35672/4988 | 1.77 (1.65 1.91) | 1.63 (1.52 1.75) |  | 35672/1549 | 1.76 (1.51 2.05) | 1.63 (1.41 1.90) |  |
| High disadvantage | 17377/3894 | 2.59 (2.37 2.82) | 2.26 (2.07 2.45) |  | 17377/1265 | 2.45 (2.07 2.90) | 2.17 (1.84 2.57) |  |
| Smoking |  |  |  | 0.033 |  |  |  | 0.085 |
| Never |  |  |  |  |  |  |  |  |
| Low disadvantage | 18583/959 | 1.00 (Ref) | 1.00 (Ref) |  | 18583/259 | 1.00 (Ref) | 1.00 (Ref) |  |
| Medium disadvantage | 29003/3409 | 1.54 (1.42 1.68) | 1.47 (1.35 1.60) |  | 29003/1213 | 1.77 (1.50 2.09) | 1.65 (1.40 1.95) |  |
| High disadvantage | 12244/2338 | 1.97 (1.78 2.17) | 1.80 (1.63 1.99) |  | 12244/836 | 2.01 (1.68 2.42) | 1.78 (1.48 2.14) |  |
| Former/Current |  |  |  |  |  |  |  |  |
| Low disadvantage | 10413/1197 | 1.00 (Ref) | 1.00 (Ref) |  | 10413/304 | 1.00 (Ref) | 1.00 (Ref) |  |
| Medium disadvantage | 21866/4325 | 1.67 (1.56 1.79) | 1.57 (1.47 1.68) |  | 21866/1259 | 1.67 (1.44 1.93) | 1.57 (1.36 1.81) |  |
| High disadvantage | 11205/3149 | 2.56 (2.36 2.78) | 2.29 (2.11 2.48) |  | 11205/972 | 2.60 (2.21 3.07) | 2.31 (1.96 2.72) |  |

Values are Hazard Ratio (HR) and 95% Confidence Intervals (CI). Social and economic disadvantage categories: Low: 0-1 disadvantage; Medium: 2-3 disadvantages; Model 1: Adjusted for sex, age, race/ethnicity, marital status, cancer, and cardiovascular disease condition. Model 2: model 1 plus social and economic factors mutually adjusted.

# Table S6. Association of combined lifestyle and SES categories with risk for all-cause and cardiovascular diseases (CVD) mortality.

| All-cause mortality | n/cases | Model 1  HR (95% CI) | Model 2  HR (95% CI) |
| --- | --- | --- | --- |
| Low disadvantage |  |  |  |
| Favorable | 14853/859 | 1.00 (Ref) | 1.00 (Ref) |
| Intermediate | 10074/797 | 1.25 (1.12 1.38) | 1.25 (1.13 1.39) |
| Unfavorable | 4069/500 | 1.84 (1.63 2.09) | 1.79 (1.58 2.03) |
| Medium disadvantage |  |  |  |
| Favorable | 19200/2246 | 1.53 (1.40 1.67) | 1.52 (1.39 1.66) |
| Intermediate | 20149/3149 | 2.09 (1.93 2.26) | 2.03 (1.87 2.20) |
| Unfavorable | 11520/2339 | 2.99 (2.74 3.26) | 2.81 (2.57 3.07) |
| High disadvantage |  |  |  |
| Favorable | 6153/1100 | 2.01 (1.82 2.23) | 2.03 (1.84 2.25) |
| Intermediate | 10000/2318 | 2.74 (2.50 3.00) | 2.69 (2.45 2.94) |
| Unfavorable | 7296/2069 | 4.43 (4.03 4.87) | 4.06 (3.69 4.47) |
| CVD mortality | n/cases | Model 1  HR (95% CI) | Model 2  HR (95% CI) |
| Low disadvantage |  |  |  |
| Favorable | 14853/220 | 1.00 (Ref) | 1.00 (Ref) |
| Intermediate | 10074/206 | 1.29 (1.04 1.60) | 1.30 (1.05 1.61) |
| Unfavorable | 4069/137 | 2.06 (1.63 2.60) | 1.95 (1.55 2.47) |
| Medium disadvantage |  |  |  |
| Favorable | 19200/753 | 1.79 (1.51 2.12) | 1.76 (1.49 2.09) |
| Intermediate | 20149/1056 | 2.49 (2.11 2.93) | 2.36 (2.01 2.78) |
| Unfavorable | 11520/663 | 2.99 (2.53 3.54) | 2.70 (2.28 3.19) |
| High disadvantage |  |  |  |
| Favorable | 6153/376 | 2.23 (1.84 2.71) | 2.18 (1.80 2.64) |
| Intermediate | 10000/813 | 3.18 (2.68 3.78) | 2.97 (2.50 3.52) |
| Unfavorable | 7296/619 | 4.61 (3.82 5.55) | 3.98 (3.31 4.79) |

Values are Hazard Ratio (HR) and 95% Confidence Intervals (CI). Social and economic disadvantage categories: Low: 0-1 disadvantage; Medium: 2-3 disadvantages; High: 4-5 disadvantages. Lifestyle categories: Favorable 0-1 risk factors; Intermediate: 2 risk factors; Unfavorable: 3-4 risk factors. Model 1: Adjusted for sex and age. Model 2: model 1 plus race/ethnicity, marital status, cancer, and cardiovascular disease condition.
